# Allergic conjunctivitis: a comprehensive review of the literature

**DOI:** 10.1186/1824-7288-39-18

**Published:** 2013-03-14

**Authors:** Mario La Rosa, Elena Lionetti, Michele Reibaldi, Andrea Russo, Antonio Longo, Salvatore Leonardi, Stefania Tomarchio, Teresio Avitabile, Alfredo Reibaldi

**Affiliations:** 1 Department of Pediatrics, University of Catania, Via S. Sofia 78, Catania, 95123, Italy; 2 Department of Ophthalmology, University of Catania, Via S. Sofia 78, Catania, 95123, Italy

**Keywords:** Allergy, Conjunctivitis, Diagnosis, Symptoms, Treatment

## Abstract

Ocular allergy represents one of the most common conditions encountered by allergists and ophthalmologists. Allergic conjunctivitis is often underdiagnosed and consequently undertreated. Basic and clinical research has provided a better understanding of the cells, mediators, and immunologic events, which occur in ocular allergy. New pharmacological agents have improved the efficacy and safety of ocular allergy treatment. An understanding of the immunologic mechanisms, clinical features, differential diagnosis, and treatment of ocular allergy may be useful to all specialists who deal with these patients. The purpose of this review is to systematically review literature underlining all the forms classified as ocular allergy: seasonal allergic conjunctivitis, perennial allergic conjunctivitis, vernal keratoconjunctivitis, atopic keratocongiuntivitis, contact allergy, and giant papillary conjunctivitis.

## Introduction

Allergic diseases have dramatically increased in the last decades [1-4]. Ocular allergy represents one of the most common ocular conditions encountered in clinical practice. A single cause of this increase cannot be pinpointed and experts are therefore considering the contribution of numerous factors, including genetics, air pollution in urban areas, pets, and early childhood exposure [5]. The associated costs have increased substantially as more of the population require treatment for allergies [6]. Ocular allergy can itself produce irritating symptoms and severe forms, such as atopic keratoconjunctivitis, could finally lead to visual loss.

Allergic conjunctivitis is an inclusive term that encompasses seasonal allergic conjunctivitis (SAC), perennial allergic conjunctivitis (PAC), vernal keratoconjunctivitis (VKC), and atopic keratocongiuntivitis (AKC). However, AKC and VKC have clinical and pathophysiological features quite different from SAC and PAC, in spite of some common markers of allergy [7]. Also contact lenses or ocular prosthesis associated giant papillary conjunctivitis (GPC) are often included in the group of ocular allergy, however they should not be considered as real allergic diseases, but as chronic ocular micro-trauma related disorders, which need to be managed by ophthalmologists in association with contact lenses experts [8].

An understanding of the immunologic mechanisms, clinical features, differential diagnosis, and treatment of ocular allergy may be useful to all specialists who deal with these patients. To this aim, we systematically reviewed literature underlining all the forms classified as ocular allergy.

## Allergic conjunctivitis

### Seasonal and perennial allergic conjunctivitis

Seasonal allergic conjunctivitis (SAC) and perennial allergic conjunctivitis (PAC) are the most common forms of ocular allergies. Estimates vary, but these types of allergy are said to affect at least 15–20% of the population [9]. The presence of specific IgE antibodies to seasonal or perennial allergen can be documented in almost all cases of SAC and PAC [10].

Allergic conjunctivitis is caused by an allergen-induced inflammatory response in which allergens interact with IgE bound to sensitized mast cells resulting in the clinical ocular allergic expression. The pathogenesis of allergic conjunctivitis is predominantly an IgE-mediated hypersensitivity reaction. Activation of mast cells induces enhanced tear levels of histamine, tryptase, prostaglandins and leukotrienes. This immediate or early response lasts clinically 20–30 min.

Mast cell degranulation also induces activation of vascular endothelial cells, which in turn expresses chemokines and adhesion molecules such as intercellular adhesion molecule (ICAM), vascular cell adhesion molecule (VCAM). Other chemokines secreted include regulated upon activation normal T cell expressed and secreted (RANTES) chemokines, monocyte chemoattractant protein (MCP), interleukin (IL)-8, eotaxin, macrophage inflammatory protein (MIP)-1 alpha.

These factors initiate the recruitment phase of inflammatory cells in the conjunctival mucosa, which leads to the ocular late-phase reaction [11,12].

**Figure 1:**
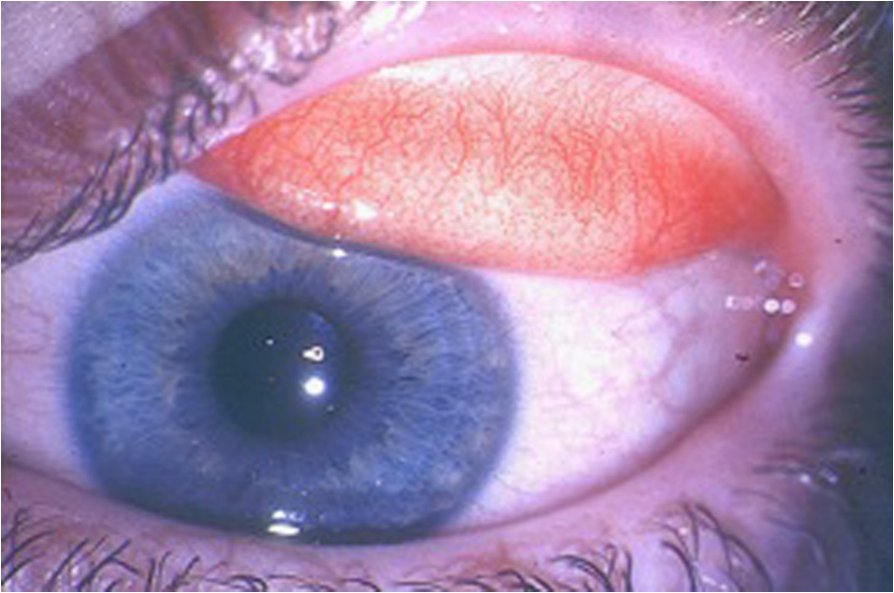
Seasonal and perennial allergic conjunctivitis: mild conjunctival injection and moderate chemosis.

Signs and symptoms of the two conditions are the same. The difference is the specific allergens to which the patient is allergic. SAC is usually caused by airborne pollens. Signs and symptoms usually occur in the spring and summer, and generally abate during the winter months. PAC can occur throughout the year with exposure to perennial allergens. Diagnostic features of SAC and PAC consist of itching, redness, and swelling of the conjunctiva. Redness, or conjunctival injection, tends to be mild to moderate (Figure 1). Conjunctival swelling, or chemosis, tends to be moderate, and somewhat more prominent than one would expect for a mild amount of redness. Itching is a fairly consistent symptom of SAC and PAC. Corneal involvement is rare [6].

### Vernal keratoconjunctivitis

VKC is a disease of warm climates and warm weather months [13,14]. It is more common in the tropics than in northern climates. However, it is not unusual to see occasional vernal conjunctivitis patients throughout the United States and Canada. The prevalence of VKC in Europe ranges from 1.2 to 10.6 cases per 10,000 population, although the prevalence of associated corneal complications is much lower (0.3-2.3 per 10,000 population) [15]. Young people are typically affected [16]. In this form, a nonspecific hyperreactivity occurs that explain the ocular symptoms induced by nonspecific stimuli – such as wind, dust and sunlight – as well as their variability, which is not related to allergen levels in the environment. Indeed, skin tests and/or serum IgE antibody tests to common allergens are often negative [13].

VKC is a chronic allergic inflammation of the ocular surface mediated mainly by Th2-lymphocyte; in a complex pathogenesis have a role also the over-expression of mast cells, eosinophils, neutrophils, Th2-derived cytokines, chemokines, adhesion molecules, growth factors, fibroblast and lymphocytes. IL-4 and IL-13 are involved in the formation of giant papillae by inducing the production of extra-cellular matrix and the proliferation of conjunctival fibroblasts [11,17,18]. VKC has three clinical forms: palpebral, limbal, and mixed, with an overall preponderance in males.

**Figure 2:**
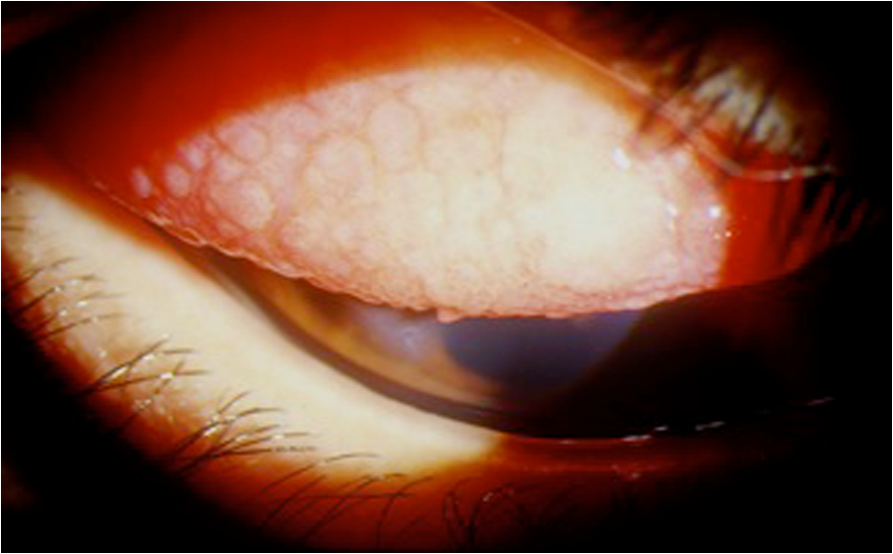
Vernal keratoconjunctivitis: giant papillae of the upper tarsal conjunctiva.

Symptoms include ocular itching, redness, swelling and discharge. Itching may be quite severe, and even incapacitating. Patients have often photophobia, sometimes very severe. The most characteristic sign is giant papillae on the upper tarsal conjunctiva (Figure 2). These ‘cobblestone-like’ swellings may be several millimeters in diameter. Usually, 10–20 are found on the tarsal conjunctiva, and they can be seen easily by ‘flipping’ the upper eyelid [7].

**Figure 3:**
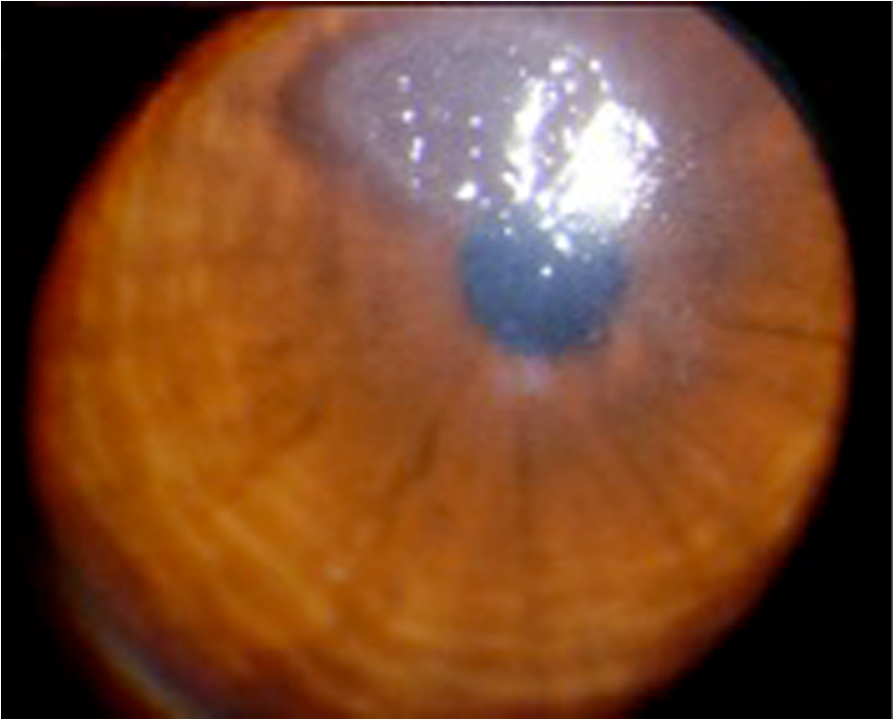
Vernal conjunctivitis: corneal plaque.

**Figure 4:**
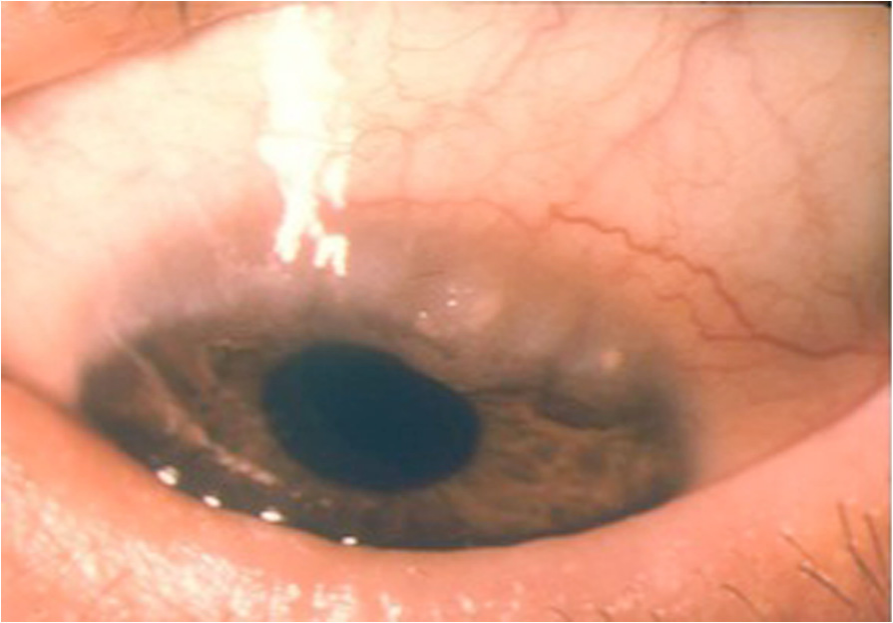
Vernal conjunctivitis: trantas dots.

There may be a tenacious mucous discharge between the giant papillae. As one might expect, the giant papillae are filled with inflammatory cells and edema. Neutrophils, plasma cells, mononuclear cells, and eosinophils are found in abundance. There is also a great deal of mast cell activity within the giant papillae. Mast cells may also be found in the conjunctival epithelium, a location in which they are not normally present. The tears of VKC patients contain high levels of IgE and mast cell mediators [19,20]. Histamine, leukotrienes, prostaglandins, and kinase may be found in the tears of VKC patients. The cornea may be affected in VKC. A punctate keratitis, known as keratitis epithelialis vernalis of El Tobgy, may begin in the central corneal. The dots may coalesce to form syncytial opacity. This often leads to a whitish or grayish plaque beneath the epithelium (Figure 3). These vernal plaques may interfere with vision and lead to central scarring of the cornea. Plaques can be removed by superficial keratectomy, but they rarely resolve without surgical intervention. Histologically, plaques consist of mucin and epithelial cells, which are literally ground into the central cornea. Tranta’s dots consist of clumps of necrotic eosinophils, neutrophils, and epithelial cells. The dots represent almost pure collections of eosinophils (Figure 4) [14]. These cells collect in crypts, which are formed by invaginations at the junction of the cornea and conjunctiva. Trantas dots tend to appear when VKC is active, and disappear when symptoms abate [6].

Shield ulcers can occur in the superior sectors of the cornea; these are noninfectious, oval-shaped circumscribed epithelial ulcer with underlying stromal opacification. After the ulcer heals, an anterior stromal opacity can persist.

The massive eosinophil infiltration and activation in the conjunctiva is responsible for the corneal complications. Corneal epithelial punctate keratitis may evolve to macroerosion, ulcers and plaques, which are all expressions of epithelial toxicity extricated by epitheliotoxic factors released by activated eosinophils [8].

### Atopic keratoconjunctivitis

Atopic keratoconjunctivitis (AKC) is a bilateral chronic inflammatory disease of the ocular surface and eyelid. Its pathomechanism involves both a chronic degranulation of the mast cell mediated by IgE, and immune mechanisms mediated by Th1- and Th2-lymphocyte derived cytokines. Also eosinophils and other inflammatory cells play a role [10,11]. It is considered the ocular counterpart of atopic dermatitis, or atopic eczema [21].

**Figure 5:**
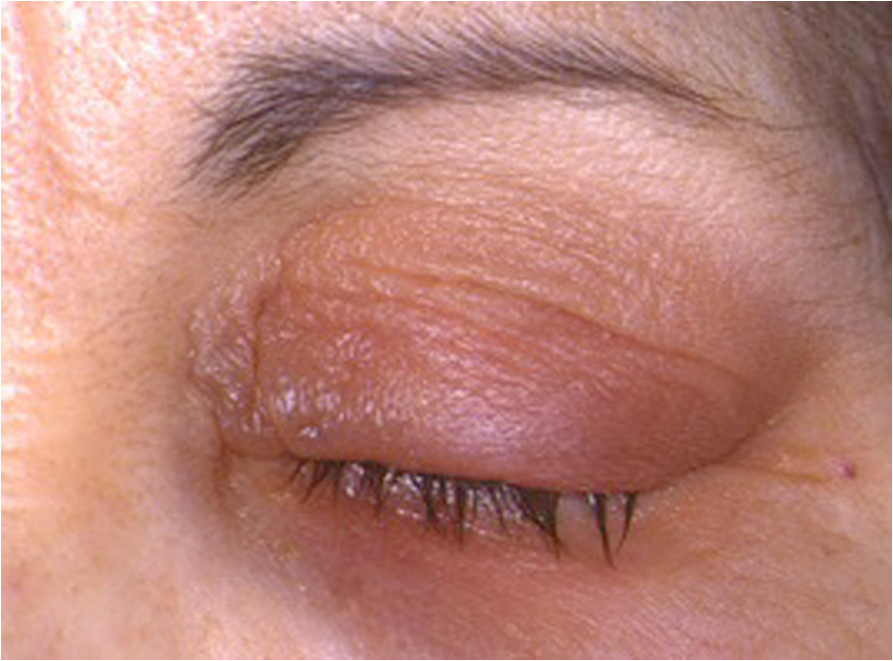
Atopic keratoconjunctivitis.

Eczematous lesions may be found on the eyelids, or any place on the body. Skin lesions are red and elevated. They often occur in the antecubital or popliteal regions. Typically, eczematous lesions are itchy, and scratching them makes them itchier. Ocular findings vary. The eyelid skin may be chemotic with a fine sandpaper-like texture (Figure 5). There may be mild, or severe, conjunctival injection and chemosis [22]. Giant papillae may, or may not, be present. Conjunctival scarring is common. Trantas dots may also be present. AKC patients may also develop atopic cataracts. Typically, these are anterior, shield-like cataracts, but nuclear, cortical and even posterior subcapsular cataracts may develop. Corticosteroid therapy in AKC patients may contribute to cataract development [22]. However, atopic cataracts were documented long before corticosteroids were available for medical use. It is not unusual for AKC patients to have cataract surgery at a young age [23]. It may seem that the appearance of VKC and AKC is similar. Both may be associated with giant papillae and Trantas dots. In fact, there probably is some overlap between these two conditions. VKC, however, resolves by age 20 years, whereas AKC can persist throughout life [6]. Many patients with AKC (45%) are skin test o allergosorbent test negative to common allergens.

### Contact allergy

Contact allergy, or allergic contact dermatitis, is not an IgE-mediated allergy, and can be considered in a different category than the before mentioned allergic conditions [24].

It is a type-IV delayed hypersensitivity response, that occurs through interaction of antigens with Th1 and Th2 cell subsets followed by release of cytokines [25].

It consists of two phases: sensitization (at the first exposition to the allergen, with production of memory T-lymphocytes), and elicitation of the inflammatory response (at the re-exposure to the antigen, mediated by the activation of memory allergen-specific T-lymphocytes).

In particular, in the sensitization phase, antigen presenting cells processed antigen-MHC class II complex interacts with T-lymphocytes, resulting in the differentiation of CD4+ T-lymphocyte into memory T-lymphocyte. In the elicitation phase, the interaction between the antigen-MHC-II complex and memory T-cells stimulates the proliferation of T-cells. The memory T-lymphocytes during proliferation release cytokines [26].

Th1 or Th2 derived cytokines perform different functions.

Th1 derived cytokines, such as IL-2, IL-3, IFN-γ, mediates recruitment of macrophages. Th2 derived cytokine, such as IL-4 and IL-5, participates in the activation and chemotaxis of eosinophils [27,28]. Two novel Th cell subsets, IL-17-producing Th cells (Th17 cells) and regulatory T cells (Treg cells) are also found to be contributors in the pathogenesis of conjunctivitis. However, the role of these cells in the activation of mast cells has not been identified clearly [29].

Allergens are generally simple chemicals, low molecular weight substances that combine with skin protein to form complete allergens. Examples include poison ivy, poison oak, neomycin, nickel, latex, atropine and its derivatives.

Contact allergy involves the ocular surface, eyelids and periocular skin,

Although contact allergic reactions usually occur on the skin, including the skin of the eyelids, the conjunctiva may also support contact allergic reactions. Initial sensitization with a contact allergen may take several days. Upon re-exposure to the allergen, an indurated, erythematous reaction slowly develops. The reaction may peak 2–5 days after re-exposure. The delay in development of the reaction is due to the slow migration of lymphocytes to the antigen depot. The term ‘delayed hypersensitivity’ is sometimes given to these reactions, in contrast to ‘immediate hypersensitivity’, a term which emphasizes the rapid development of IgE antibody-mediated reactions. Contact allergic reactions are generally associated with itching. Treatment consists of withdrawing, and avoiding contact with allergen. Severe reactions can be treated with topical or systemic corticosteroids [6].

### Giant papillary conjunctivitis

**Figure 6:**
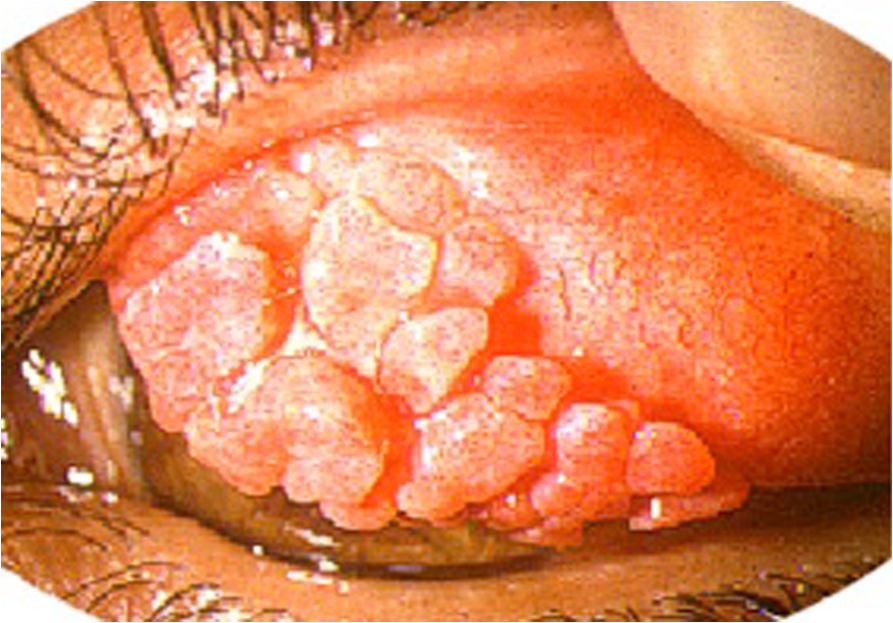
Giant papillary conjunctivitis.

Giant papillary conjunctivitis (GPC) is an inflammatory disease characterized by papillary hypertrophy of the superior tarsal conjunctiva; the appearance is similar to vernal conjunctivitis [30], but there is no significant corneal involvement (Figure 6).

GPC is not an allergic disease; the incidence of systemic allergy in GPC patients is similar to that of the general population, and the stimuli for the papillary conjunctival changes are inert substances rather than allergens. For example, GPC may be caused by limbal sutures, contact lenses, ocular prostheses, and limbal dermoids [31]. When these irritative stimuli are removed, the conjunctival papillary changes resolve. The conjunctival tissues may contain mast cells, basophils, or eosinophils, but not to the extent of an allergic reaction. There is no increase in IgE or histaimine in the tears of GPC patients. Since the advent of disposable contact lenses, the frequency of GPC is low.It appears that protein build-up on the surface of contact lenses, and irregular edges were the main reason for the close association between contact lenses and GPC [6], by immune or mechanical mechanisms: in particular protein deposits on the surface of the contact lens could become antigenic and stimulate the production of IgE; mechanical trauma and chronic irritation can determine the release of some mediators (CXCL8 and TNF-α) from injured conjunctival epithelial cells [9,32].

## Diagnosis of allergic conjunctivitis

The diagnosis of ocular allergy is primarily clinical, but there are laboratory tests that can be useful in supporting the diagnosis [33]. Allergists can perform skin testing for specific allergens by scratch tests or intradermal injections of allergen. In-vitro tests for IgE antibodies to specific allergens are widely used. Allergic tests would help in differentiating intrinsic and extrinsic forms and would, therefore, be helpful in the treatment [6].

## Treatment options

Avoidance of the offending antigen is the primary behavioral modification for all types of allergic conjunctivitis; however, the eyes present a large surface area and thus it is often impossible to avoid ocular exposure to airborne allergens. Artificial tear substitutes provide a barrier function and help to improve the first-line defense at the level of conjunctival mucosa. These agents help to dilute various allergens and inflammatory mediators that may be present on the ocular surface, and they help flush the ocular surface of these agents. When avoidance of non-pharmacologic strategies do not provide adequate symptom relief, pharmacologic treatments may be applied topically or given systemically to diminish the allergic response.

The mainstay of the management of ocular allergy involves the use of anti-allergic therapeutic agents such as antihistamine, multiple action anti-allergic agents and mast cell stabilizers. For example, the H_1_ topical antihistamine levocabastine hydrochloride is effective in rapidly relieving ocular inflammation when administered topically to the eye [34,35]. Topical antihistamines competitively and reversibly block histamine receptors and relieve itching and redness but only for a short time. These medications do not affect other proinflammatory mediators, such as prostaglandins and leukotrienes, which remain uninhibited. A limited duration of action necessitates frequent dosing of up to 4 times per day, and topical antihistamines may be irritating to the eye, especially with prolonged use [36]. Combination treatments using decongestants with antihistamines have been shown to be more effective, and are administered to the eye as drops up to 4 times daily [37]. Decongestants act primarily as vasoconstrictors and are effective in reducing erythema, however, adverse effects include burning and stinging on instillation, mydriasis, and rebound hyperemia or conjunctivitis medicamentosa with chronic use [37]. Therefore, these treatments are suitable only for short-term symptom relief, and are not recommended for use in narrow-angle glaucoma patients.

Mast cell stabilizers have a mechanism of action that is unclear. They may increase calcium influx into the cell preventing membrane changes and/or they may reduce membrane fluidity prior to mast cell degranulation. End result is a decrease in degranulation of mast cells, which prevents release of histamine and other chemotactic factors that are present in the preformed and newly formed state.

Mast cell stabilizers do not relieve existing symptoms and they can be used on a prophylactic basis to prevent mast cell degranulation with subsequent exposure to the allergen. Mast-cell stabilizing medications can also be applied topically to the eye, and may be suitable for more severe forms of conjunctivitis. They require a loading period during which they must be applied before the antigen exposure. Therefore, poor compliance should be taken into account as a possible drawback.

In recents years have been introduced several multimodal anti-allergic agents, such as olopatadine, ketotifen, azelastine and epinastine and bepostatine, that exert multiple pharmacological effects such as histamine receptor antagonist action, stabilization of mast-cell degranulation and suppression of activation and infiltration of eosinophils [38].

Ketotifen inhibits eosinophil activation, generation of leukotrienes and cytokine release [39,40].

Azelastine is a selective second generation H1 receptor antagonists, and also acts by inhibiting platelet activating factor (PAF) and blocking expression of intercellular adhesion molecule 1 (ICAM-1) [41]. Epinastine has effect on both H1 and H2 receptors (the latter effect may be beneficial in reducing the eyelid swelling), and also has mast-cell stabilizing and anti-inflammatory effects [42].

These drugs are becoming the drug of choice for providing immediate symptomatic relief for patients with allergic conjunctivitis.

When the abobe mentioned anti-allergic drugs do not allow an adequate control of the allergic inflammatory process, anti-inflammatory agents are used. Non-steroidal anti-inflammatory drug (NSAIDs) can be used as additive drugs, in order to,reduce the conjunctival hyperemia and the pruritus, related in particular to prostaglandin D2 and prostaglandin E2 [43].

Corticosteroids remain among the most potent pharmacologic agents used in the more severe variants of ocular allergy and are also effective in the treatment of acute and chronic forms of AC [44-48]. Corticosteroids possess immunosuppressive and anti-proliferative properties since they can hinder the transcription factor that regulates the transcription of Th2-derived cytokine genes and differentiation of activated T-lymphocytes into Th2-lymphocytes. They have some limitations, including ocular adverse effects, such as delayed wound healing, secondary infection, elevated intraocular pressure, and formation of cataract. These agents are therefore appropriate for short courses (up to 2 weeks); however, if needed for longer durations, an eye examination should be carried out, including baseline assessment of cataracts and intraocular pressure measurement [3,49].

The efficacy of immunotherapy against ocular symptoms precipitated by conjunctival antigen challenges was originally demonstrated in 1911 and this well-established method may be considered for the long-term control of AC [50]. Although some more recent studies have focused on nasal rather than ocular symptoms, others have confirmed the efficacy of immunotherapy against ocular symptoms [50-56].

Allergen-specific immunotherapy is an effective treatment for patients with allergic rhinoconjunctivitis that have specific IgE antibodies to allergens. The main objective of this treatment is to induce a clinical tolerance to the specific allergen: it reduces the seasonal increases of IgE specific for that allergen, and it increases the production of specific IgG4 and IgA; such effects are mediated by an increase of the production of IL-10 and TGF-β1 [57].

However, immune responses to allergen administration are not predictive of the effectiveness of the therapy and the therapy itself can produce systemic reactions, the incidence and severity of which vary dependent on the type of allergen administered [58,59]. Traditionally, immunotherapy is delivered via subcutaneous injection. However, sublingual (oral) immunotherapy (SLIT) is gaining momentum among allergists. SLIT requires further evaluation for ocular allergy relief; it has been shown to control ocular signs and symptoms, although ocular symptoms may respond less well than nasal symptoms [60-65]. Oral antihistamines are commonly used for the therapy of nasal and ocular allergy symptoms. These newer second-generation antihistamines are recommended in preference to first-generation antihistamines because they have a reduced propensity for adverse effects such as somnolence [3]. Second-generation antihistamines can, however, induce ocular drying, which may impair the protective barrier provided by the ocular tear film and thus actually worsen allergic symptoms [66,67]. It has therefore been suggested that the concomitant use of an eye drop may treat ocular allergic symptoms more effectively [67]. Intranasal corticosteroids are highly effective for treating nasal symptoms of allergic rhinitis, but the evidence that they may also be effective for the treatment of ocular symptoms is inconsistent [68-70].

## Pediatric allergic conjunctivitis

In the pediatric age, allergic conjunctivitis occurs frequently, with a peak age in late childhood and young adulthood. Patients frequently have a history of other atopic disease, such as eczema, asthma, or, most commonly, rhinitis. Symptoms include bilateral involvement, itching, tearing, mucoid discharge, redness, mild eyelid edema, and chemosis. AKC and VKC occur less commonly, but are potentially more severe. Therefore, involvement of pediatric ophthalmologists may be necessary to avoid preventable vision loss in severe cases [71].

## Conclusion

The term allergic conjunctivitis is an inclusive term that encompasses different clinical entities based on the assumption that the classical Type I hypersensitivity mechanism is responsible for all clinical forms of allergic eye disease. However, IgE and non-IgE-mediated mechanisms are involved in the development of ocular allergic diseases. The multiple mediators, cytokines, chemokines, receptors, proteases, growth factors, intracellular signals, regulatory and inhibitory pathways, and other unknown factors and pathways are differently expressed in the different allergic disorders, inducing the different clinical aspects, diagnostic features and response to treatment. Therefore, a new classification system is desirable, preferably derived from the varied pathophysiological mechanisms operating in the different forms of ocular allergy.

### Consent

Written informed consent was obtained from the patient for publication of this report and any accompanying images

## Competing interests

The authors declare that they have no competing interests.

## Authors’ contributions

ML, and AR have made substantial contributions to conception and design of the review, interpretation of data, and revising the manuscript critically for important intellectual content; EL, and MR, have made substantial contributions to conception and design of the review, acquisition of data and analysis, interpretation of data, and drafting the manuscript; AR, ST, and GV have made substantial contributions to acquisition of data and analysis, and drafting the manuscript. All authors have given final approval of the version to be published.
